# Meeting of Minds around Food Addiction: Insights from Addiction Medicine, Nutrition, Psychology, and Neurosciences

**DOI:** 10.3390/nu12113564

**Published:** 2020-11-20

**Authors:** Aymery Constant, Romain Moirand, Ronan Thibault, David Val-Laillet

**Affiliations:** 1 INRAE, INSERM, University Rennes, NuMeCan, Nutrition Metabolisms Cancer, 35590 St Gilles, 35000 Rennes, France; aymery.constant@ehesp.fr (A.C.); romain.moirand@chu-rennes.fr (R.M.); ronan.thibault@chu-rennes.fr (R.T.); 2 EHESP, School of Public Health, 35043 Rennes, France; 3 Unité d’Addictologie, CHU Rennes, 35000 Rennes, France; 4 Unité de Nutrition, CHU Rennes, 35000 Rennes, France

**Keywords:** obesity, craving, reward circuit, motivation, cognition, behavior, therapy

## Abstract

This review, focused on food addiction (FA), considers opinions from specialists with different expertise in addiction medicine, nutrition, health psychology, and behavioral neurosciences. The concept of FA is a recurring issue in the clinical description of abnormal eating. Even though some tools have been developed to diagnose FA, such as the Yale Food Addiction Scale (YFAS) questionnaire, the FA concept is not recognized as an eating disorder (ED) so far and is even not mentioned in the Diagnostic and Statistical Manuel of Mental Disorders version 5 (DSM-5) or the International Classification of Disease (ICD-11). Its triggering mechanisms and relationships with other substance use disorders (SUD) need to be further explored. Food addiction (FA) is frequent in the overweight or obese population, but it remains unclear whether it could articulate with obesity-related comorbidities. As there is currently no validated therapy against FA in obese patients, FA is often underdiagnosed and untreated, so that FA may partly explain failure of obesity treatment, addiction transfer, and weight regain after obesity surgery. Future studies should assess whether a dedicated management of FA is associated with better outcomes, especially after obesity surgery. For prevention and treatment purposes, it is necessary to promote a comprehensive psychological approach to FA. Understanding the developmental process of FA and identifying precociously some high-risk profiles can be achieved via the exploration of the environmental, emotional, and cognitive components of eating, as well as their relationships with emotion management, some personality traits, and internalized weight stigma. Under the light of behavioral neurosciences and neuroimaging, FA reveals a specific brain phenotype that is characterized by anomalies in the reward and inhibitory control processes. These anomalies are likely to disrupt the emotional, cognitive, and attentional spheres, but further research is needed to disentangle their complex relationship and overlap with obesity and other forms of SUD. Prevention, diagnosis, and treatment must rely on a multidisciplinary coherence to adapt existing strategies to FA management and to provide social and emotional support to these patients suffering from highly stigmatized medical conditions, namely overweight and addiction. Multi-level interventions could combine motivational interviews, cognitive behavioral therapies, and self-help groups, while benefiting from modern exploratory and interventional tools to target specific neurocognitive processes.

## 1. Introduction

Even though the concept of food addiction (FA) was introduced more than sixty years ago [1], its definition and implications are still fiercely debated [2,3]. Highly palatable foods [4,5], such as processed foods with added sugars and fat, could be as addictive as drugs [6,7], acting via the same neurocognitive and hedonic processes [8,9]. Therefore, through the alteration of the neurocognitive systems involved in food intake control [10], FA could be involved in the obesity pathogenesis. However, at this time, the concept of FA is still debated [2,3,11] and is probably entangled with complex psychological factors and predispositions. The concept of sugar addiction is well defended in animal models [12], but in humans, some authors rather suggest the concept of “eating addiction”, i.e., an addiction to the eating behavior instead of an addiction to palatable foods like sugar or saturated fat. Nevertheless, considering the alterations of the neurocognitive systems involved in food intake control [10], the diagnostic criteria of FA were based on the Diagnostic and Statistical Manuel of Mental Disorders version 5 (DSM-V) criteria for substance use disorders [13]. In clinical practice, the Yale Food Addiction Scale (YFAS) 2.0 is the only self-administered questionnaire validated to diagnose and estimate the number of symptoms of FA [14,15].

Our first aim is to better describe the place of FA in the current nosography and establish a parallel between FA and other ED or addictive disorders, while discussing possible transfer or continuity between disorders. Our second aim is to illustrate, on the basis of existing data, how FA is frequent in the general and obesity population and how it articulates with comorbidities in obese patients. We also discuss why and how FA should be handled in the preoperative management of obesity surgery patients. Our third aim is to highlight the relationship between FA and specific psychological features, within a continuum ranging from normal to disordered eating. Our fourth aim is to describe the neurocognitive and brain correlates with other substance use disorders (SUD), such as with drugs and alcohol, to support a neurobiological picture of FA. Finally, we discuss the concept of FA in the context of prevention, diagnostic, and treatment, with the aim to present existing or innovative strategies in the scope of interdisciplinary and personalized medicine. Perspectives in terms of cognitive and behavioral therapies, digital technologies, and neuromodulation interventions are also discussed.

### 1.1. From the Addiction Medicine Clinician Point of View: Towards a Definition of Food Addiction (FA)

Addiction remains a difficult-to-define concept. The American Society of Addiction Medicine defined addiction as “a treatable, chronic medical disease involving complex interactions among brain circuits, genetics, the environment, and an individual’s life experiences. People with addiction use substances or engage in behaviors that become compulsive and often continue despite harmful consequences” [16]. DSM-5 (Table 1) and ICD-11 give similar sets of criteria defining addiction, while actually avoiding using the term, instead preferring “substance-related and addictive disorders” (including “substance use disorders” (SUD) and “gambling disorder”) and “dependence”, respectively. These criteria encompass the loss of control over consumption, increased motivation to consume, and persistent consumption despite negative consequences, as well as tolerance or adverse effects of acute withdrawal. In the last twenty years, there has been a growing interest in the possibility that in some patients, food, and especially highly palatable food, could produce behavioral symptoms that parallel those of addiction and could activate the same neural reward circuits as drugs of abuse [17]. However, this concept has been challenged, either on the addictive nature of some eating disorders (ED) [18,19] or by debating as to whether FA is akin to a behavioral addiction versus a SUD [20].

Diagnostic criteria for SUD represent a cluster of cognitive, behavioral, and physiological symptoms, and most of them can apply to some patients by replacing “substance” with “certain food” (for extensive reviews, see [4,7,21]). “Taking larger amounts of the substance for longer periods than intended” has been cited as one of the most commonly reported symptoms in overweight/obese or eating disorder patients. It can be in the form of binges, but also snacking [22], food compulsion, or excessive portion sizes. “Unsuccessful attempts to reduce food intake” is clinically obvious, as many patients are unable to maintain their diet and lose weight in the long term. “Craving” is a central concept in the field of addiction [23], and craving for food has been recognized for a long time [24,25,26]. Similarity between drug and food craving is supported by the findings of cue-reactivity research [24]. “Social/interpersonal problems related to use” is supported by the poor social functioning associated with overweight and obesity due to weight stigmatization. Negative consequences of overeating include obesity and its medical consequences, stigmatization, psychological distress induced by shame, hopelessness [27]; then, many patients exhibit “continuous use despite recurrent physical or psychological problem”. These negative consequences lead to the “failure to fulfill major role obligation” and to “reduced activities”. Tolerance can be suspected, given that some overweight patients increased consumption and portion size over time [21]. This was further supported by an innovative study in which 61 overweight carbohydrate-craving women were induced into a sad mood, then exposed, double-blind and in counterbalanced order, to taste-matched carbohydrate or protein beverages and asked to choose the drink that made them feel better. They overwhelmingly chose and liked the carbohydrate beverage, which was more efficient in reducing dysphoria, but the effect decreased with repetition, suggesting tolerance, while liking increased, suggesting sensitization [28]. At last, a specific food withdrawal syndrome, as can be observed with alcohol, opioids, or nicotine, has not clearly been demonstrated in humans. However, it should be noted that the physiological criteria of tolerance and withdrawal are not necessary for a diagnosis of SUD, as even with a potent substance such as alcohol, withdrawal syndrome is observed in no more than one third of patients.

Gearhardt and coworkers have boosted studies on FA by releasing the Yale Food Addiction Scale (YFAS), by transposing the DSM-IV [29], then DSM-5 (YFAS 2.0) [30] criteria for substance dependence, then SUD, simply by replacing substance with “certain food” in the criteria. In comparison to the first version of the YFAS, the YFAS 2.0 explores the following additional criteria, the first three issued from the previous DSM-IV diagnostic of abuse: continued consumption despite social or interpersonal problems, failure to fulfill major role or obligation, use in physically hazardous situations, and craving. YFAS 2.0 comprises 35 questions exploring the 11 criteria of DSM-5 SUD and the existence of significant impairment or distress and uses exactly the same thresholds to assign an FA diagnosis (Table 1). Of note, DSM-5 allows us to grade SUD as mild, moderate, and severe, according to the number of criteria, and only severe SUD fits well with the diagnosis of dependence in DSM-IV and ICD-11. In other words, sensitivity was increased, and it is questionable to say that a mild SUD responds to the criteria of addiction [31]. Numerous systematic reviews have explored validation, prevalence, and correlates of FA diagnosed by the YFAS [4,15,32,33,34,35]. Gordon et al. [4] evaluated empirical studies examining the construct of “FA” and their conclusions supported FA as a unique construct consistent with criteria for other SUD diagnoses. The highest scored symptom was generally “unsuccessful attempt to cut down”; tolerance and “use despite knowledge of adverse consequences” were also frequent, followed by “activities given up” and withdrawal symptoms [35]. In studies using YFAS 2.0, severe FA predominated upon mild and moderate forms [35]. YFAS number of symptoms was correlated with body mass index (BMI) in non-clinical samples; this was variable in obese or ED samples [35]. Significant positive correlations were found between FA and depression or anxiety [32,35]. FA, in obese patients or university students, was consistently associated with self-report and other measures of impulsivity; associations with reward sensitivity were inconsistent, depending on the questionnaires used [34]. At last, FA prevalence was generally increased in patients with other chemical or behavioral addictions as compared with non-clinical samples [36,37,38,39,40].

The triggering mechanism of FA has been extensively debated. Hebebrand et al. [20] have argued that FA may be a behavioral addiction: indeed, most substances of abuse, apart from alcohol, are agonists of specific brain receptors by mimicking endogenous ligands, and this is not applicable to food. Moreover, eating is necessary for survival and drug use is not; eating is intrinsically rewarding and reinforcing, and food consumption is well known to naturally activate the brain reward system. Most other authors, however, considered FA to fit better with SUD [4,7,13,37,41,42]. Two properties of food could participate in mediating liking, wanting, or craving: hedonic taste and metabolic shifts following ingestion. Food contains a variety of compounds that may serve as chemical or metabolic triggers, and all commonly suspected problem foods share nutritive properties [41]. It is highly unlikely that all foods may be addictive, and studies have aimed at identifying the specific foods or food attributes capable of triggering an addictive response. The evidence that sugar (sucrose) could be an addictive substance is mainly supported by animal studies, the interpretations of which are controversial [18,42,43], and sugar (sucrose, fructose) is not considered a direct cause of obesity [42]. In university students, symptoms of FA were in majority related to combined high-fat savory and high-fat sweet foods, and rarely for mainly sugar-containing food [2]. Concerning fat, which has its own metabolic, physiological, and nutritional profiles, human evidence is scarce and comes mostly from studies on FA: individuals with FA had higher dietary fat intake compared to those without FA; animal models suggested that fat addiction may have different mechanisms to sugar addiction [44]. Highly processed foods, with the addition of fat and/or refined carbohydrates (sugar, white flour), have drawn the most attention. Subjects’ rating of food pictures varying in their chemical composition showed that highly processed, energy dense foods with high glycemic load and high fat content were most frequently associated with addiction-like eating behaviors, especially for individuals endorsing elevated symptoms of FA [5]. Another study showed that high processed food pictures were associated with greater loss of control, liking, pleasure, and craving, which assess the abuse liability of substances [45]. This was confirmed using a taste test task and ad libitum consumption period in obese women (39% had an FA diagnosis) [46]. Highly processed foods with high glycemic index cause rapid shifts in blood glucose, insulin, and other metabolic fuel and hormones [41], and this could be associated with their addictive potential.

The concept of FA has many implications for the addiction clinician. Given the prevalence of FA in people with addiction, screening for FA and other eating disorders has to be performed in patients with other addictive disorders. FA could explain, by a mechanism of addiction transfer, the increase in the consumption of chocolate and other sweets in recovering patients with alcohol use disorder [47] as well as the tragic increase in alcohol use disorders after obesity surgery [48]. Denial is a well-recognized, albeit badly explained, feature of patients with SUD [49], but has not been explored in the FA literature. Given the importance of social stigma attached both to overweight and addiction [50], it is very likely that denial does exist, particularly in obese/overweight samples seeking treatment. Such a behavioral feature could minimize FA prevalence. Denial is in part related to concerns about being stigmatized or rejected or to social interactions of an accusatory or judgmental nature [49]. There have been extensive discussions about the influence of the recognition of the FA construct on stigmatization, either from family/relatives and society (externalized stigma) or from the patient himself (internalized stigma) [50,51]. An FA explanation model of the lack of control in obesity could decrease stigmatization from others [52]. Studies on the effects on self-esteem and internalized stigmatization gave contradictory results [50,53]. Increasing self-esteem and confidence could then constitute a therapeutic goal in obesity treatment.

In conclusion, the analogy between severe SUD and some eating-related behaviors including FA is obvious from a clinical point of view, although more studies are needed to precisely determine the triggering mechanisms and the possibility of preventive or therapeutic interventions.

### 1.2. From the Clinical Nutritionist’s Point of View: Food Addiction (FA) in the Context of Obesity Treatment

In this section, we aim to: (i) illustrate how FA is frequent in the general and obese population and how it articulates with comorbidities in obese patients; (ii) discuss why and how FA should be handled in the management of obese patients, especially those referred to obesity surgery.

**Figure 1:**
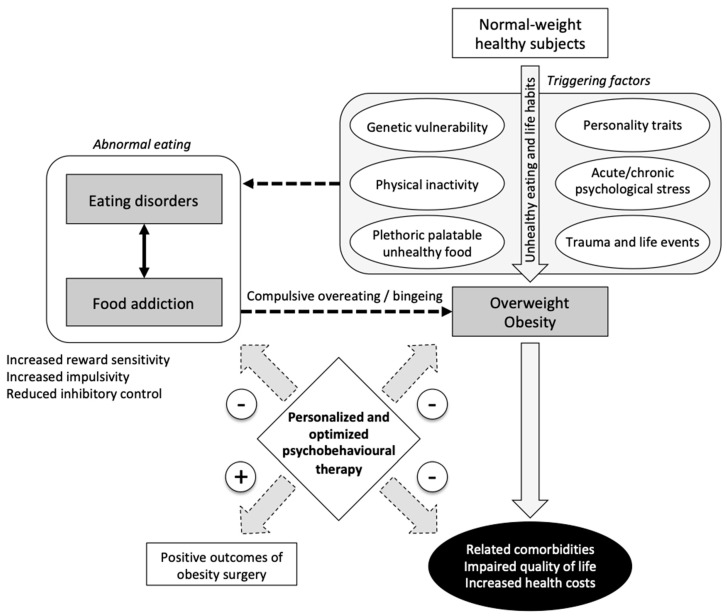
Food addiction as a causative or contributive factor for overweight and obesity. A personalized and optimized psychobehavioral therapy in patients with food addiction may help in preventing overweight and obesity, reducing their related comorbidities and related costs, and improving outcomes of obesity surgery. Dotted lines indicate connections for which published data are lacking or insufficient.

Obesity is pandemic worldwide [54,55] and leads to well-known comorbidities [56], representing a significant socioeconomic burden [57,58,59] (Figure 1). Obesity medical treatments usually fail to achieve weight loss or maintain it in the long term [60], justifying the recourse to obesity surgery in some instances. Obesity surgery decreases mortality, cardiovascular events, and type-2 diabetes in comparison to conventional therapy [60,61,62,63]. Nevertheless, 20% to 30% of operated patients regain weight because of the reoccurrence of ED, i.e., binge eating disorders, hyperphagia, snacking, craving, food compulsion, or bulimia [64]. These symptoms could be related to FA.

As previously mentioned, highly palatable foods [4,5], such as processed foods with added sugars and fat, could be as addictive as drugs [6,7], acting via the same neurocognitive and hedonic processes [8,9]. Therefore, through the alteration of the neurocognitive systems involved in food intake control [10], FA could be involved in obesity pathogenesis (Figure 1). As there is currently no validated therapy against FA, FA is often underdiagnosed and untreated [13,19]. FA may partly explain the failure of obesity treatment.

#### 1.2.1. Prevalence of FA in the General and Obese Population

FA is frequent in the general and obese populations. Through meta-analysis, the mean prevalence of FA diagnosis was found to be 16.2%, more frequent in obese/overweight patients (from 10% in normal weight to around 25% in people with obesity), with the greatest prevalence in patients with ED [15,32]. FA was more frequent in women than in men [35]. In the US general population, Schulte and Gearhardt [65] reported that 15% of people may have FA, regardless of BMI. Pursey et al. [15] found that 19.9% of overweight and obese patients had FA. Som et al. [66] found a large prevalence (almost 40%) of FA in patients eligible for obesity surgery. The analysis of 19 studies which assessed FA among pre- and/or post-obesity surgery patients revealed that the presence of pre-surgical FA was not associated with pre-surgical weight or post-surgical weight outcomes; yet pre-surgical FA was related to broad levels of psychopathology [33]. The prevalence of FA has been reported to be 16.5% [67], 17.2% [68], 25% [69], or 40% [66,70] in obese patients referred to obesity surgery. Prevalence could be even higher in the case of ED [25,71]: 57% in patients with bulimic hyperphagia [72], 41% [72,73] and up to 96% in patients with binge-eating disorders patients and bulimia, respectively [74]. Pursey et al. found that FA prevalence was higher in overweight/obese patients (24.9%) than in subjects with normal weight (11.1%) [15], in accordance with other findings [12,13,65]. Kiyici et al. found that 32% of obese patients with a mean BMI of 41.6 and seeking treatment for weight loss had FA [75]. 

#### 1.2.2. Association between Food Addiction and Obesity-Related Comorbidities

It remains unclear whether FA could be associated with or even favor obesity-related comorbidities. Kiyici et al. found that fasting plasma glucose level was lower in patients with FA, but serum insulin levels, homeostasis model assessment of insulin resistance, hemoglobin A1c, lipid parameters, and vascular adiposity index were comparable [75]. In obese patients with BMI ≥35 referred to obesity surgery, FA was not associated with obesity-related complications, such as cardiovascular diseases including arterial hypertension, obstructive sleep apnea syndrome (OSAS), type-2 diabetes, disabling osteoarticular disease, or non-alcoholic steatohepatitis [66]. Overall, FA could be considered as a potential contributing factor leading to obesity, but not to its complications, which are also driven by metabolic, environmental, or genetic factors.

#### 1.2.3. Rationale for a Systematic Screening of FA in Obese Patients

Given the high prevalence of FA (almost 40% of patients referred to obesity surgery), evaluating FA should be part of the assessment of any obese patient, especially in patients referred to obesity surgery, as done for ED. However, there is no official recommendation about FA. Binge eating disorder and bulimia nervosa are a contraindication to obesity surgery because they increase the risk of postoperative complications, such as vomiting, esophagus dilation [76,77], addiction transfer [78], or weight regain. In children, FA in relation to psychological trauma was associated with a reduced likelihood of completing obesity surgery [79]. Only a few studies have looked at the relationship between FA and the success of behavioral or surgical obesity therapy. Pepino et al. [70] suggested that obesity surgery-induced weight loss induces remission of FA and improves several eating behaviors that are associated with FA. Lent et al. [80] reported that baseline FA status was not associated with weight loss 6 months after medical intervention in 178 adult obese patients. In a small sample size of 57 overweight or obese patients, but followed-up only for seven weeks, Burmeister et al. [81] found less weight loss in the case of FA. In morbidly obese patients, Som et al. did not find any relationship between baseline FA and weight loss in response to behavioral therapy [66]. Sevinçer et al. [60] found that the prevalence of FA of 58% in the preoperative period decreased significantly after obesity surgery to 7% and 14% at 6 months and 1 year, respectively. 

Whereas obesity surgery could be beneficial for FA [70,82], there is an increased risk of “addiction transfer” from FA to another one. Indeed, obese patients may be at increased risk for SUD after obesity surgery [83]. The proportion of new substance users (alcohol, smoking, or drugs) after obesity surgery ranged from 34.3% to 89.5% [84]. Up to 20% of obese patients are diagnosed with alcohol use disorder after obesity surgery [85]. New-onset alcohol use disorder can represent more than 60% of alcohol use disorder in obese patients after obesity surgery [48]. This is why it is fundamental to diagnose FA and ED before obesity surgery. Future studies should demonstrate whether individualized cognitive behavioral therapy dedicated to the management of FA should prevent the occurrence of addiction transfer and optimize the postoperative outcomes after obesity surgery, especially in terms of the prevention of postoperative ED and weight regain [76,78]. For example, a recent review was aimed at assessing the outcomes of preoperative and post-operative psychosocial interventions for bariatric surgery patients, revealing mixed evidence but also the importance of acting early, before significant problematic eating behavior and weight regain occur [86].

We could also suggest studies assessing whether the YFAS 2.0 questionnaire could be integrated into obese patient phenotyping. If relevant, this could be included in the Edmonton Obesity Staging System (EOSS) [85,87] or French Obesity Staging System (FOSS) [88], which already integrate the psychological dimension. Limitations for the diagnosis of ED and FA are the evaluation by patient declaration, which could lead to potential information bias related to self-assessment objectivity or honesty. The proportion of patients with FA could be underestimated, as it is for all declarative information. This is why the development of complementary diagnostic strategies is required, in terms of biological, neurological, or behavioral markers, for example.

#### 1.2.4. Proposed Therapy for Obese Patients with FA

So far, no psychological therapy has been validated in the management of FA in obese patients. In our opinion, this should be included in multidisciplinary programs combining empathic approaches and social support interventions, to help patients in coping with their daily life struggles and social stigma. In our center (Nutrition Department, CHU Rennes), all obese patients with BMI ≥35 are able to lose weight by following a one-year psychobehavioral program, including six multidisciplinary consultations (nutritionist physician, dietician, and nurse specialized in therapeutic education), in order to manage or prevent ED. This program includes at each meeting with the patient: motivational interview, advice for physical activity, and food education. In addition, all patients have two psychological consultations to help them consider the importance of emotions and stress in their eating behavior, but they are not psychotherapy sessions. All the patients expected to be eligible for obesity surgery participate in this program for an average of one year before obesity surgery.

In conclusion, as the prevalence of FA is high in obese patients, especially in patients referred for obesity surgery (40%), it is a relevant issue for the clinical practice of obesity care. Future studies should assess whether dedicated management of FA is associated with better outcomes, especially after obesity surgery. Given the prevalence of FA in the general population, public health policies should help in screening early and managing FA before it leads to obesity, which is a burden worldwide (Figure 1).

### 1.3. From the Health Psychologist’s Point of View: Toward a More Comprehensive Psychological Approach to Food Addiction

Contrary to the categorical approach of the medical or psychiatric practices, psychology considers that eating behaviors can be mapped onto a continuum ranging from normal to disordered eating, prompted by multiple environmental, contextual, and individual factors [89,90]. For instance, environments constantly influence unhealthy food choices and overeating through food cues—sights, sounds, and smells—associated with palatable food [91], which may undermine the self-regulatory capacity in obesogenic environments [92]. It is recognized that problematic eating behaviors—such as binge eating episodes, overeating, and (failed) cognitive restriction—are not limited to psychological disorders and tend to increase over time in the general population [93].

Studies have found that 7.2% to 13% of the population currently engage in regular binge eating episodes [94]. Another study found that their prevalence increased six-fold from 1998 (2.7%) to 2015 (13.0%) in the adult general population [95]. In addition to these binge eating episodes, eating in response to specific emotional cues was investigated in relation to weight gain [96,97], ED [98,99], and psychiatric and addictive disorders [100,101]. However, this behavioral response is common in normal-weight women, as half of the female students participating in our study reported overeating in response to anxiety in the last 28 days, and 4 in 10 in response to loneliness, sadness, and happiness [102]. These intermittent overeating episodes were used as a time-limited response to emotional states and negatively correlated with alcohol use, which suggests two distinct and somewhat exclusive ways of coping for negative emotions. The Three-Factor Eating Questionnaire Revised, 18-item (TFEQ-R18), measures the cognitive and behavioral components of eating [103], which originate from obesity research but are present in other populations. It includes three subscales: (1) Cognitive Restraint (conscious restriction of food intake in order to control body weight or to promote weight loss) comprised of six items (e.g., “I consciously hold back at meals in order not to gain weight”), (2) Uncontrolled Eating (tendency to eat more than usual due to a loss of control over intake accompanied by subjective feelings of hunger), comprised of nine items (e.g., “When I see a real delicacy, I often get so hungry that I have to eat right away”), and (3) Emotional Eating (inability to resist emotional cues), comprised of three items (e.g., “When I feel blue, I often overeat”). In our study, inability to resist emotional cues outweighed other cognitive components of eating which, again, suggests that overeating is a common tendency to cope with negative emotions. Moreover, while problematic eating behaviors were initially approached independently, they may interact and/or co-exist in complex patterns.

In many cases, overeating may be a paradoxical consequence of attempts at caloric restriction [104,105], and overlaps exist with emotional overeating and binge eating episodes, as studies showed a direct relationship between binge eating disorder (BED), stress, anxiety, and anxiety proneness [106,107]. However, outside of bulimia nervosa studies, much of the theoretical and empirical binge eating research to date has not directly addressed the role of anxiety [108]; even less has addressed the role of other emotional states such as depression, boredom, or fatigue. Personality may also have a structural albeit overlooked role in problematic eating. For instance, a recent study provided a phenotypic characterization of the FA construct by conducting a clustering analysis of FA in patients with eating disorder and obesity [109]. They found the highest FA symptoms in the “dysfunctional clusters”, characterized by more dysfunctional personality traits, greater impulsivity, and more general psychopathology. Conversely, the “adaptive” cluster presented with more functional personality traits and low levels of general psychopathology, as well as the lowest levels of FA. This suggests that FA in the adaptive cluster may be the result of different factors than in other clusters, which could have important implications for treatment. Another study showed that emotional eating was strongly positively associated with neuroticism, particularly impulsiveness and depression [110]. External eating was likewise mainly associated with the characteristics of impulsiveness (e.g., tendency to act impulsively under strong negative and positive affective experiences, to act on the spur of the moment without regard for the consequences, to enjoy activities that are exciting or novel, etc.) and lower self-discipline [111]. Restrained eating was, on the other hand, related to higher conscientiousness, extraversion and openness, and lower neuroticism. These results imply that poor self-control seen in impulsiveness and lower self-discipline was most important for eating due to negative emotions as well as in response to external food stimuli. Attempts to control food intake and body weight seen in restrained eating were associated with more character strengths and ambitions and also a more outgoing personality style with more stable emotions.

In this regard, the lack of mental stimulation could constitute a significant vulnerability factor for excessive eating [112] and drinking [113]. Som et al. found a higher proportion of food addiction in unemployed patients [66], and our previous work showed a greater predisposition to boredom in patients with excessive drinking [113]. One possible explanation is that some vulnerable people use these compulsive behaviors to cope with excessive lack of internal and/or external stimulation in their daily lives, which may increase the risk of addiction and jeopardize their social and professional functioning. This fits with the definition of one of the eleven diagnostic criteria of addiction in the DSM-5: giving up important social, occupational, or recreational activities because of substance use. Secondly, FA includes the negative feelings following compulsive eating, typically guilt and shame, which are also commonly reported amid overeating episodes in the general population [95]. One possible explanation is that negative feelings after overeating episodes come from the social stigma attached to weight issues [114] rather than from the overeating episode itself. This emotional response to internalized weight stigma could explain the high proportion of FA diagnosis in obese patients, although, in most cases, obesity is the result of poor dietary habits rather than compulsive eating [105,115]. Accordingly, a large part of the FA syndrome, as assessed by the YFAS 2.0, could be seen as a context-dependent pattern of problematic eating behaviors and negative feeling, existing in various forms and intensity, in the general population as well as in patients with chronic conditions, independently of any psychiatric disorders. Finally, tolerance and withdrawal are the only symptoms specific to addictive processes in FA, since they are unrelated to environmental and individual factors and therefore possibly those distinguishing FA from highly frequent problematic eating behaviors. This would be consistent with a review on FA [4], concluding that behavioral and substance-related aspects of FA appear to be intertwined, but the substance (highly palatable food) component may be more salient to the diagnostic classification of this phenomenon than the behavior (eating). 

From this perspective, pharmacological criteria, namely craving towards palatable food and withdrawal symptoms, could constitute the main—if not the only—solid indicators of FA, possibly co-existing with varying patterns of problematic eating behaviors, negative feelings, and social disturbances. Investigating their relative contributions to FA, together with their interactions with social environments, unhealthy eating habits, and clinical outcomes, could contribute greatly to the understanding of FA developmental history. However, the YFAS conception study adopted confirmatory factor analysis (CFA), a theory-based approach that can only estimate the extent to which questionnaire data fit the theoretical single-factor structure derived from DSM-5 criteria. The main advantage of CFA lies in its ability to help researchers to bridge the frequent gap between theory and observation. One disadvantage of CFA is that secondary factor loadings are not part of the output [116]. This may lead to the assumptions that (1) all items belong to the same single-factor variable, by simply ignoring the other possible structural hypotheses, and (2) they are equally important for characterizing FA, even though their relative contributions (loadings) to the latent variable are quite heterogeneous in most studies. It must be noted that elevated loadings are expected in construct validation studies since summary scores (or, in the present case, symptoms scores) are computed for clinical or research purposes [117]. Most analyses were performed on the 11 binary diagnoses instead of the original 7-point Likert items, which limited the total variance to be analyzed. This approach is clearly suboptimal in the case of a newly devised instrument, being psychometrically investigated for the first time. Consequently, the extent to which each of these behavioral, psychological, and social disturbances contribute to FA is still unclear. Unraveling these complex relationships warrants data-driven approaches that establish the data’s underlying structure by addressing a wide range of candidate hypotheses, i.e., exploratory factor analyses. This could allow for a more comprehensive description of FA as a biopsychosocial construct lying on a continuum from normal to disordered eating and therefore earlier identification of high-risk profiles.

### 1.4. From the Behavioral Neuroscientist’s Point of View: Is There a “Food Addict” Brain?

Before answering the question “is there a ‘food addict’ brain?”, it is necessary to remember how drug addiction is described in light of its brain phenotype. As stated earlier, the DSM-5 [118] does not recognize FA in itself, but it identifies different forms of substance-related and addictive disorders (including gambling) that can be used as a reference framework for our discussion. In this context, if we accept the existence of FA, then the neurobiological characteristics of substance-related and addictive disorders should reveal common patterns between food and drug abuse. Valuable recent review papers were aimed at describing common underlying neurobiological mechanisms contributing to drug and FA [119,120]. One of the main pitfalls of these overviews, which is honestly highlighted by the authors but often bypassed in general, is the fact that most human studies taken as support were performed in obese subjects and/or patients suffering from eating disorders (ED), especially bingeing ED-subtype patients. This bias is usually accepted because there are very few studies that aimed at characterizing the brain phenotype/responses of human patients who have been specifically diagnosed with FA. There is consequently a significant risk for circular reasoning: because we make the assumption that FA should resemble drug addiction and its associated brain phenotype, then the observation of this specific brain phenotype in obese and/or bingeing patients should be sufficient to defend the FA hypothesis. The point is that the FA construct must be supported by precise definitions, as well as dedicated neurobiological and neuroimaging studies. These definitions must be supported by concrete data and not only by shortcuts based on analogies with obesity or food abuse. Even though we must assume that substance addiction always starts with substance use, not all obese and/or bingeing ED-subtype patients have FA, and not all “food addicts” are obese.

The diagnosis of an SUD is based on a pathological pattern of behaviors related to use of the substance, and the DSM-5 assists this diagnosis with eleven criteria categorized under four groupings (Table 1). It is important to mention that only one criterion, related to craving (Criterion 4), refers to a specific brain pattern associated with this condition. Craving corresponds to an intense desire or urge for the substance and is described as being associated with the activation of specific reward structures in the brain.

The literature on the neurobiology of addiction provides a consensus on the fact that drugs of abuse, as well as particular excessive behavioral patterns (e.g., gambling), exert a direct activation of the brain reward system. On a chronic basis, they also induce profound neuronal plasticity changes in the corticostriatal and limbic systems. Initially, drugs of abuse trigger abnormal surges of dopamine in the nucleus accumbens, which promotes the direct striatal pathway and inhibits the indirect striato-cortical pathway [121]. Repeated drug consumption and/or administration induce mesolimbic sensitization [122], as well as neuroplasticity changes in the glutamatergic inputs to the striatum and midbrain dopamine neurons. These changes enhance the brain’s reactivity to drugs and their associated cues that gain incentive salience, i.e., incentive sensitization [123], reduce the sensitivity to other types of reward, decrease cognitive control mechanisms, and increase the susceptibility to stress and emotional dysregulation [121]. Eventually, there is a transition between controlled to habitual and compulsive use or intake [124]. The precise neuropharmacological mechanisms involved in this transition may depend on the type of drugs used, but a recurrent feature of repeated exposure to substances of abuse is the downregulation of the dopaminergic system, especially the dopamine type-2 receptor (D2R) in the ventral and dorsal striatum. Similar observations have been made in humans [125] and animal models [126], but this is not the scope of our review. Here, we are rather interested in the very few studies that tried to describe the brain phenotype of patients who were specifically diagnosed with FA.

As stated by Fletcher and Kenny [18], information will be lost if we begin with the assumption that drug addiction processes explain food overconsumption and schedule our empirical endeavors exclusively toward a survey of similarities, some of which are superficial and imprecise. In light of the DSM-5 substance use nosology, many authors consider FA as a true addiction [13,127]. As previously stated, the most widely used and accepted tool to measure FA to date is the Yale Food Addiction Scale (YFAS), of which Version 2.0 has been validated in different languages in addition to English [30], including French, Spanish, and Japanese [101,128,129]. As a consequence, we decided to gather information on brain imaging studies that were aimed at describing anatomical and functional features that are characteristic of patients fitting the YFAS criteria for FA (Table 2).

The first study of this kind was performed by Gearhardt herself in collaboration with American colleagues from different teams investigating the neural correlates of eating behavior [136]. These authors used the well-known “milkshake paradigm” of blood-oxygen-level-dependent (BOLD) functional magnetic resonance imaging (fMRI) in response to receipt and anticipated receipt of palatable food (i.e., chocolate milkshake). With this paradigm, it has been well described that obese compared to lean individuals show greater activation of the gustatory cortex and oral somatosensory regions in response to anticipated intake and consumption of palatable foods. Obese individuals also show increased activation in the orbitofrontal cortex and putamen in response to palatable food pictures (i.e., reward anticipation), as well as decreased activation in the caudate nucleus in response to consumption of milkshake vs. a tasteless solution (i.e., reward receipt) [137,138]. Gearhardt et al. [136] demonstrated that YFAS scores correlated with greater activation in the anterior cingulate cortex, orbitofrontal cortex, and amygdala in response to anticipated receipt of food. Subjects with higher vs. lower YFAS showed greater activation of the dorsolateral prefrontal cortex and caudate, in response to anticipated receipt of food, but less activation in the lateral orbitofrontal cortex in response to receipt of food. This enhanced anticipation of the rewarding properties of food resembles the reward surfeit theory of obesity, suggesting that individuals at risk for obesity initially show hyper-responsivity of reward circuitry to high-calorie food cues, which would further increase intake of such foods. The fact that some of the reward circuit responses are decreased after consumption of the palatable food illustrates a reward deficit that may drive further intake to fulfill the need for food pleasure. The loss of control over food intake is an important criterion for FA. This inhibitory control was specifically investigated in youth with symptoms of FA by Hardee et al. [130] using a dedicated go/no go task. They demonstrated that YFAS-positive subjects showed deactivation in three clusters of brain regions including the middle temporal gyrus/occipital gyrus, precuneus/calcarine sulcus, and inferior frontal gyrus. The inferior frontal gyrus, notably, has been regularly described as being involved in executive and motor control, and decreased activation of this structure is usually interpreted as a lack of inhibitory control during a go/no go task. The decreased activity in the other clusters was perhaps related to decreased sustained attention during the task; a lack of attention, notably towards interoceptive perceptions, might contribute to the difficulty of obese people to regulate calorie intake, as postulated by Volkow et al. [139]. These results are somewhat corroborated by another study demonstrating that people who meet the YFAS criteria for FA have impaired performance monitoring, both on the behavioral and neural levels, consequently sharing some neurocognitive characteristics with patients diagnosed with substance use disorder [135].

Surprisingly, Gearhardt et al. [136] found no correlation between YFAS scores and BMI, which suggests that FA can occur in subjects within different body weight categories (as confirmed by the prevalence data previously cited). Even though the authors showed limited differences in reward circuitry activation between high- and low-YFAS subjects during food intake, high-YFAS individuals exhibited patterns of neural activation associated with reduced inhibitory control, which might explain their difficulty to resist food craving. In our opinion, two main questions arise from this work, and they still require additional retrospective and prospective studies to provide answers. First, does a FA profile in normal-weight individuals increase the risk to further declare obesity or other nutritional disorders, as postulated by the reward surfeit theory? Second, since almost all imaging studies performed in obese subjects did not include the YFAS score as a factor in their analysis, what is the probability that the brain pattern associated with YFAS in some “undiagnosed” individuals had influenced the general patterns observed in obese people? Considering the high prevalence of FA in obese patients, there is a significant bias in most studies describing the brain patterns characteristic of obesity, simply because there are many forms and behavioral phenotypes of obesity. Most studies probably characterized brain responses in obese subjects with different clinical profiles, since YFAS was not part of their routine checking, and a fair proportion of their obese subjects might very well have been YFAS-positive. Consequently, this percentage of “undiagnosed” YFAS patients might have influenced and biased our knowledge of the “obesity brain phenotype”.

Interestingly, Beyer et al. [131] showed that symptoms of FA were not associated with the major structural brain differences correlated with BMI in the general population, but they might rather explain additional variance towards a lower right lateral orbitofrontal cortex thickness. Whether this anatomical specificity in the orbitofrontal cortex is responsible for the functional differences observed in this particular structure after food reward [136] necessitates further validation. However, the criteria for manifest FA were met by only 6% of the general population in this study [131], which does not indicate what the effects of YFAS symptoms on brain anatomy would be in a large cohort exclusively composed of obese subjects with higher prevalence of FA. 

Using (18) F-2-fluoro-2-deoxyglusose (18FDG) positron emission tomography (TEP) instead of BOLD fMRI, Guzzardi et al. [133] investigated in overweight women the brain responses to high-calorie sweet food pictures and found greater activation in the thalamus, hypothalamus, midbrain, putamen, and occipital cortex, but not in the prefrontal and orbitofrontal cortices, in high-YFAS compared to low-YFAS subjects. Interestingly, in high-YFAS women, metabolic responsiveness in the orbitofrontal cortex was progressively lower with increasing YFAS severity and hunger subjective ratings. The authors’ conclusions were that inadequate activation in response to the rewarding food in brain regions involved in inhibitory control and reward processing, in spite of greater activation in brain areas involved in somatosensory stimuli processing, reward and memory of hedonic behavior, distinguishes overweight women with FA from women with similar overweight but not FA [133]. It is also very interesting to highlight that the same authors demonstrated that a 3-month low-calorie diet was sufficient to reverse these specific brain activation patterns, which suggests that weight loss (3.8 kg or 4.1% of initial body weight in high YFAS) can help in correcting the neurocognitive anomalies associated with FA [133], exactly as demonstrated for the brain anomalies associated with obesity in formerly obese women who have successfully lost weight [140]. However, restrictive diets are usually ineffective in the long term and should not be advocated alone as obesity treatment.

The nutritional environment consequently has a major role in sustaining or correcting brain anomalies related to FA. The regular consumption of palatable high-calorie foods profoundly modifies many cognitive processes related to food perception, valuation, and motivation. The incentive sensitization theory postulates an excessive amplification of the psychological “wanting” of food, but it also highlights the particular role of external triggering cues and specific attention to these cues in maintaining a vicious circle. Food cue reactivity was found to be modified in overweight or obese women with YFAS-diagnosed FA, towards modest, elevated responses in the superior frontal gyrus for highly processed food pictures and more robust, decreased activations for minimally processed food cues, these responses being opposite in control subjects with similar overweight or obesity [132]. Exteroceptive stimuli such as visual cues are therefore very important in triggering and maintaining the neurocognitive patterns of food addiction. As reminded by Gearhardt et al. [136], activation in the nucleus accumbens is associated with craving in SUD and the amygdala is commonly implicated in drug cue reactivity and craving. Interestingly, Osadchiy et al. [134] demonstrated in healthy subjects with or without elevated BMI that YFAS scores had positive associations with functional connectivity between the amygdala and nucleus accumbens. In the same study, gut microbiota-derived indole metabolites were found to have a direct positive association with BMI and an indirect positive association with YFAS through functional connectivity of the nucleus accumbens [134], which might suggest a role of the gut microbiota in hedonic food intake in the context of FA. Both exteroceptive (e.g., related to food and environment) and interoceptive cues (e.g., related to the internal state and gut microbiota metabolites) are consequently important to understand how the neurocognitive patterns of FA emerge and establish in the long term, with the possibility to increase the risk for further psychological and metabolic disorders.

**Figure 2:**
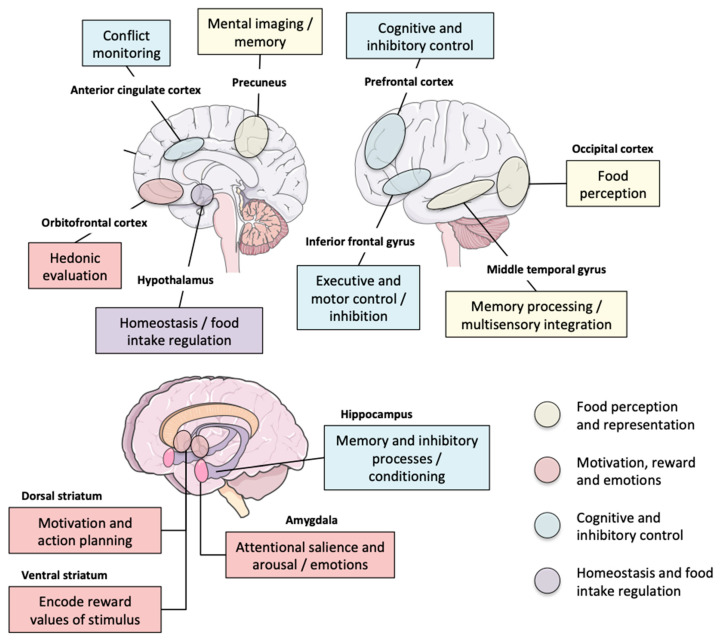
Neurocognitive functions and brain areas that are impacted by food addiction and for which people who meet the YFAS criteria for food addiction have different brain activity, metabolism, or functional connectivity compared to normal subjects. Please refer to Table 2 for details on results and imaging modalities used. Brain schematic representations were collected from Servier Medical Art (Suresnes, France; http://www.servier.fr).

All these data support the existence of a specific FA brain phenotype that can be detected in normal-weight, overweight, or obese individuals and that is characterized by anomalies in the reward and inhibitory control processes, with likely corollary consequences in the limbic/emotional and cognitive/attentional spheres (Figure 2). Even though a recent meta-analysis of fMRI studies defends an addiction model of obesity, characterized by reduced cognitive control and interoceptive brain responses [141], this vision is probably restricted to part of the obesity spectrum and cannot be generalized to all forms of obesity. Further research is needed to better phenotype the neurobehavioral patterns of YFAS-positive subjects and disentangle their complex relationships and overlap with other diseases including obesity and other forms of addiction. Such work is mandatory to improve medical care because a better understanding of the patients’ specificities leads to better treatment. As reminded by Ho et al. [142], post-obesity surgery patients are at increasing risk for developing alcohol and SUD, which likely represents an “addiction transfer” from food to other means of fulfilling the individuals’ drives for pleasure or comfort. This risk could be especially increased if the presence of an FA profile has not been diagnosed and treated beforehand. The YFAS 2.0 questionnaire is a useful tool to predict continued emotional and binge eating behavior following obesity surgery [143] and might be used to identify subpopulations of patients with higher risk for unsuccessful obesity surgery. However, as a questionnaire, this method remains limited by the usual constraints and uncertainties of declarative diagnostic methods, which necessitates the development of additional diagnostic tools and markers, derived from brain imaging or biological measurements at the gut–microbiota–brain level, for example.

## 2. General Discussion and Conclusions

The aim of this review was to enlighten the current concept of FA through four different angles and the spectrum of complementary disciplines: addiction medicine, nutrition in the context of obesity management, health psychology, and behavioral neurosciences. In our opinion, only a multidisciplinary perspective can render the complexity of FA and how it relates to environmental, social, and individual factors, while being inscribed in a continuum ranging from normal to disordered eating. This multifactorial comprehension of FA is needed to better organize prevention, diagnostic, and treatment, through the implementation of existing but also future strategies in the scope of personalized medicine. Even though the concept of personalized medicine sometimes appears hackneyed nowadays, it is particularly important and relevant in the context of FA and obesity treatment, considering the variety of individual profiles or situations, as well as the complex combination of environmental and individual factors at their origin.

Evaluation of FA is very unusual during first-line medical management and is not even systematic during obesity consultation. Practices are highly variable and dependent on the medical services and clinicians in charge of these consultations. As mentioned earlier, there is still no recognized psychological therapy validated for the management of FA in obese and/or ED patients, even though these patients represent high-risk profiles for FA. The role of FA in favoring body weight management problems and related comorbidities is still unclear, but early detection and treatment of FA might prevent the onset of further medical problems. The use of the YFAS questionnaire, when disordered eating is suspected, should become widespread in general medicine and specialized consultations, before referring the patient to a person with expertise in the management of ED and FA. This could be facilitated by the existence of a short version which is easier to fill. Because questionnaire studies are always subject to the biases and limitations of declarative methods, and denial could be present in some patients, there is a need for objective markers for which modern neuroimaging might represent an asset. Other biological markers might be explored—for example, at the metabolome and gut microbiota levels [134]—since the relationship between the gut microbiota and some neurocognitive processes has been extensively demonstrated.

Moreover, the FA construct has important treatment implications [21,51]. The standard approach to weight loss involves maintaining a healthy diet and physical exercise and is often associated with poor adherence and success rates. In the range of existing strategies are cognitive interventions [21], psychobehavioral interviews, and counseling via medical staff specialized in therapeutic education and nutrition, but also consultations with psychotherapists or psychiatrists. Addressing the psychological impact of internalized social stigma on patients remains pivotal, as several authors raised concerns that a diagnosis of food addiction could result in a double or additive stigma [50]. This emphasizes the need for empathic approaches and social support interventions in patients’ management programs. For instance, the implementation of a self-help support group through a structured program could promote mutual support between persons with FA, break isolation, and create a space for sharing experiences [144]. Some authors also reviewed the beneficial input of online support options for food addiction, as well as other forms of self-help groups and sessions [145,146]. The restriction, or even the relative reduction, of some specific foods seen as addictive for a specific patient could be an option, contrary to the current view which aims at reducing dysfunctional dieting in favor of regular eating with flexible and moderate food consumption with no forbidden foods [51]. Such an approach is advocated by anonymous group meetings such as Overeaters Anonymous (OA), based directly on the 12-step program developed by Alcoholic Anonymous, which might help patients to break social isolation and the vicious circle at the origin of some forms of overeating [147], but we still lack perspective on the long-term success of such a strategy. Other initiatives can be applied in the context of FA, such as acceptance and commitment therapy (ACT) [148]. A wide range of motivational interviewing and cognitive behavioral therapies (CBT), which requires patients to critically evaluate the thoughts, feelings, and behaviors resulting in maladaptive responses and helps them to find their own solutions, adapted to their daily lives, can also be implemented in the context of FA and have already demonstrated their usefulness [149]. Such results must be replicated on larger cohorts and in the long term, and different types of CBT should be compared to provide recommendations about matching strategies to individual profiles, depending on their personality traits and susceptibilities, for example.

Several authors praised the use of innovative neuromodulation strategies to treat obesity, ED, but also FA [21,150], with the aim to modulate if not normalize some brain activities and neurocognitive processes involved in food intake control. If we put aside invasive strategies such as deep brain stimulation (DBS), there are still several candidates in the scope of minimally invasive strategies, such as transcranial direct current stimulation (tDCS), transcranial magnetic stimulation (TMS), and real-time fMRI neurofeedback. All these techniques can be used to stimulate or inhibit specific brain regions. The tDCS and TMS, via external electric or magnetic stimulation, are rather restricted to superficial (i.e., cortical) brain areas, such as the prefrontal cortex, which plays an important role in the cognitive control of eating. The rtfMRI can be applied to any brain area (including the deep striatal component of the reward circuit), since this method relies on the ability of the subject to voluntarily modify his/her brain activity on the basis of real-time feedback on this activity (e.g., via a visual gauge) combined with explicit or implicit tasks or mindfulness techniques. This approach has already been validated, with promising outcomes in healthy, overweight, and obese women, with the aim to reduce hunger and cravings [151,152]. 

Prevention, diagnosis, and treatment should also benefit from new developments in the scope of information and communication digital technologies. Innovative smart devices, smartphone applications, and online counseling platforms might provide potent tools for phenotyping individual profiles, adjusting eating habits on a daily basis, and providing information to both patients and actors of medical care. Preliminary data suggested the effectiveness of a mobile health app based on FA to treat young obese people [153]. The faster disordered eating and FA are detected, the easier corrective measures can be applied in order to prevent the onset of a vicious circle and complete loss of control over food intake, further leading to obesity and numerous comorbidities.

## Figures and Tables

**Table 1:** Diagnostic and Statistical Manuel of Mental Disorders version 5 (DSM-5) diagnostic criteria of substance use disorder (SUD). A person needs to meet at least 2 of these criteria and have significant impairment or distress from his pattern of substance use to be diagnosed with a SUD. The severity of addiction is determined by the number of criteria met: 2–3 mild; 4–5 moderate; ≥6 severe.

Broader Categories	SUD Criteria
Impaired control	Substance often taken in larger amounts or over a longer period than was intended Craving, or a strong desire or urge to use the substance Persistent desire or repeated unsuccessful attempts to quit and/or control substance use Great deal of time is spent in activities necessary to obtain or use the substance or recover from its effects
Social impairment	Continued use despite having persistent or recurrent social or interpersonal problems caused or exacerbated by the effects of the substance Recurrent substance use resulting in a failure to fulfill major role obligations at work, school, or home Important social, occupational, or recreational activities are given up or reduced because of substance use
Continued used despite risk	Recurrent substance use in situations in which it is physically hazardous Substance use is continued despite knowledge of having a persistent or recurrent physical or psychological problem that is likely to have been caused or exacerbated by the substance
Pharmacological criteria	Tolerance: Need for markedly increased amounts of the substance to achieve intoxication or desired effect or Markedly diminished effect with continued use of the same amount of the substance Withdrawal: Withdrawal syndrome (differs by substance) or Substance is taken to relieve or avoid withdrawal symptoms

**Table 2:** Studies investigating the brain anatomical or functional specificities associated with YFAS-diagnosed food addiction (FA) in the human.

Articles Titles	Subjects	Exploration Methods	Main Results	References
Neural correlates of inhibitory control in youth with symptoms of FA	76 young subjects (8.2–17.8 yo, 44 males)	Go/no-go task during BOLD fMRI	YFAS-positive subjects showed deactivation in three clusters: middle temporal gyrus/occipital gyrus, precuneus/calcarine sulcus, and inferior frontal gyrus	[130]
Neuroanatomical correlates of food addiction symptoms and body mass index in the general population	625 subjects (Leipzig Research Centre for Civilization Diseases LIFE-Adult study), 20–59 yo, 45% women	BMI, personality questionnaires including YFAS and TFEQ, and brain structure via high-resolution 3T MRI	Small, additional contribution of YFAS symptom score to lower right lateral orbitofrontal cortex thickness over the effect of BMI	[131]
Food cue reactivity in FA: A functional magnetic resonance imaging study	44 women with overweight or obesity, *n* = 20 with moderate-to-severe YFAS FA	YFAS, BOLD fMRI cue reactivity task	Subjects with FA exhibited modest, elevated responses in the sFG for highly processed food images and more robust, decreased activations for minimally processed food cues, whereas control subjects showed the opposite responses; Housefold items elicited greater activation than the food cues in regions associated with interoceptive awareness and visuospatial attention (e.g., INS, iFG, iPL)	[132]
FA distinguishes an overweight phenotype that can be reversed by low calorie diet	36 overweight women	YFAS, 18 FDG-PET	Greater activation in thalamus, hypothalamus, midbrain, putamen, and occipital cortex (reward), but not in prefrontal and orbitofrontal cortices (control/reward receipt) in the high-YFAS versus low-YFAS group. In high-YFAS subjects, orbitofrontal responsiveness was inversely related to YFAS severity and hunger rating, and positive associations were observed between regional brain activation and lipid intake. A 3-month low-calorie diet abolished group differences in brain activation	[133]
Correlation of tryptophan metabolites with connectivity of extended central reward network in healthy subjects	63 healthy subjects with and without elevated BMI (29 men and 34 women)	Fecal sampling, HAD anxiety and YFAS questionnaires, functional and anatomical connectivity of the amygdala, nucleus accumbens, and anterior insula	Direct positive association of indole metabolites with BMI and indirect positive association with YFAS through functional connectivity of the nucleus accumbens	[134]
FA is associated with impaired performance monitoring	34 YFAS-positive and 34 control subjects	YFAS, Eriksen flanker task, and EEG measurement	YAFS-positive subjects have reduced ERN and Pe waves and demonstrate a higher number of errors on the flanker task, suggesting impaired performance monitoring	[135]
Neural correlates of FA	49 healthy adolescent females ranging from lean to obese	YFAS, BOLD fMRI in response to receipt and anticipated receipt of palatable food (chocolate milkshake)	YFAS correlated with greater activation in the aCC, OFC, and amygdala in response to anticipated receipt of food. Participants with higher (*n* = 15) vs. lower (*n* = 11) YFAS showed greater activation in the DLPFC and CAU in response to anticipated receipt of food, but less activation in the lOFC in response to receipt of food	[136]

aCC, anterior cingulate cortex; BMI, body mass index; BOLD, blood-oxygen-level-dependent; CAU, caudate; DLPFC, dorsolateral prefrontal cortex; EEG, electroencephalography; ERN, error-related negativity; FA, food addiction; FDG, F-2-fluoro-2-deoxy-glucose; fMRI, functional magnetic resonance imaging; iFG, inferior frontal gyrus; INS, insula; iPL, inferior parietal lobe; lOFC, lateral orbitofrontal cortex; OFC, orbitofrontal cortex; Pe, error positivity; PET, positron emission tomography; TFEQ, three-factor eating questionnaire; YFAS, Yale Food Addiction Scale; yo, years old.
